# Regulatory B Cells Evaluation in Systemic Lupus Erythematosus Patients with Subclinical Atherosclerosis and Secondary Antiphospholipid Syndrome

**DOI:** 10.31138/mjr.03823.rbc

**Published:** 2023-08-03

**Authors:** Marwa Mahmoud Abdelaziz, Nihal Fathi, Helal F. Hetta, Ahmed Abdel-Galeel, Mohamed Zidan, Eman M. Shawky, Rania M. Gamal

**Affiliations:** 1 Rheumatology and Rehabilitation Department, Faculty of Medicine, Assiut University, Assiut, Egypt,; 2 Medical Microbiology and Immunology Department, Faculty of Medicine, Assiut University, Assiut, Egypt,; 3 Cardiovascular Medicine Department, Heart Hospital, Faculty of Medicine, Assiut University, Assiut, Egypt,; 4 Diagnostic Radiology Department, Faculty of Medicine, Assiut University, Assiut, Egypt

**Keywords:** systemic lupus erythematosus, subclinical atherosclerosis, secondary antiphospholipid syndrome, B-lymphocytes, Breg cells

## Abstract

**Objectives:**

The current knowledge of human studies that address B cells in Systemic Lupus Erythematosus (SLE) patients with subclinical atherosclerosis remains insufficient. We aimed to evaluate the contribution of Breg cells in SLE and secondary antiphospholipid syndrome (APS) patients taking into consideration its relation to subclinical atherosclerosis and the disease activity.

**Methods:**

Thirty SLE patients and 23 controls were included. Systemic Lupus Erythematosus Disease Activity Index-2000 was estimated. Evaluation of Breg cells percentage using flow cytometry was done. All participants underwent carotid doppler ultrasound examination for measurements of the intima-media thickness of the common carotid artery (cIMT). The coronary artery calcium scoring was calculated using the Agatston method.

**Results:**

The mean± SD of age was 32.60±8.34 years, while of the age of onset was 28.27±7.60 years. Twenty-three patients (76.7%) had subclinical atherosclerosis. There was a highly significant difference in Breg cells between SLE and APS patients with subclinical atherosclerosis and controls (P= 0.001, 0.005). SLE and APS patients had significantly higher mean cIMT than control (P*=*0.01, 0.050). Breg cells had 70% sensitivity and 87% specificity for diagnosing of SLE (P=0.01). Multivariate regression analysis indicated that low Breg cells were predictive for the disease activity (OR=1.76, 95% CI=1.21- 2.85; P= 0.01).

**Conclusion:**

SLE patients had a high frequency of subclinical atherosclerosis, those and patients with secondary APS had a high risk of plaque formation. We found a contribution of Breg cells in SLE patients with subclinical atherosclerosis. Breg cells are considered a good predictor of diagnosis of SLE.

## INTRODUCTION

Systemic lupus erythematosus (SLE), an autoimmune disorder characterised by abnormal T cells, overactive B cells, and the production of autoantibodies, could affect affecting various several organs and threaten life.^1^ Antiphospholipid syndrome (APS) is a systemic autoimmune disease of hypercoagulability characterised by liable to venous and arterial thrombosis, recurrent foetal losses, and the presence of antiphospholipid antibodies (APA).^2^ patients with SLE present have a high prevalence of early atherosclerosis and a greater considerable risk of developing cardiovascular diseases (CVD).^3^ Additionally, SLE patients with secondary APS have an elevated prevalence of carotid plaque.^4^ An early finding in SLE patients is subclinical atherosclerosis which is considered a vital predictor of CVD risk that accounts for more than one-third of SLE patients’ deaths.^5, 6^ The adaptive and innate immune systems participate in the inflammation of SLE and atherosclerosis, and therefore the development and progression of CVD. Disease duration and activity, nephritis, dyslipidaemia, autoantibodies, and circulating immune complexes are SLE-related risk factors for accelerated atherosclerosis.^7^ The endothelial damage encourages atherogenesis in SLE via the deposition of circulating immune complexes or binding of antibodies to endothelial cells.^8^ Oxidized low-density lipoprotein triggers an inflammatory and immunogenic process that encourages endothelial dysfunction and secretion of pro-inflammatory cytokines, resulting in B lymphocyte activation, autoantibody production, and formation of immune complexes that hasten the intracellular accumulation of lipids within atherosclerotic plaques.^9^ Elevated levels of circulating OxLDL and antibodies against OxLDL are more common in SLE patients with CVD.^10^ Reduced capacity to clear apoptotic cells and necrotic debris is an important factor in both diseases.^11^ It was believed that B cells are protective in atherosclerosis due to the protective autoantibodies they produce. while newer studies revealed that B cells have proatherogenic functions.^10^ This confirms the complexity of the functions of B cells, and other immune cells in both atherosclerosis and SLE.^12^ Regulatory B cells (Bregs) are B cell subsets with immunosuppressive function Bregs secrete inhibitory cytokines, like interleukin (IL)-10, IL-35, and transforming growth factor (TGF-β).^13^ Human Bregs aren’t of a single phenotype, a subset of B cells with CD19^+^CD24^high^CD38^high^ is vital in autoimmunity like SLE and atherosclerosis.^14^ Bregs create the protective role by attenuating the neointimal formation of atherosclerosis through an IL-10- mediated mechanism.^15^ The current knowledge of human studies that address B cells in patients of SLE with subclinical atherosclerosis or secondary APS remains insufficient; therefore this work evaluates the contribution of Breg cells in SLE patients with subclinical atherosclerosis and secondary APS patients taking into consideration its relation to subclinical atherosclerosis and correlation with the disease activity.

## MATERIALS AND METHODS

### Study design

We enrolled 30 adult patients with SLE who fulfilled The Systemic Lupus Collaborating Clinics (SLICC) revised and validated the American College of Rheumatology (ACR) SLE classification criteria^16^ and the diagnosis of APS (10 patients) by the revised Sapporo criteria.^2^ A control group of 23 blood donors as healthy volunteers, age- and sex-matched was included during this cross-sectional study. We recruited participants from rheumatology inpatient and outpatient clinics in the Rheumatology and Rehabilitation department in our University Hospital during the period between December 2019 and November 2020*.* Written informed consent was taken from all participants. The study design was approved by the Scientific Ethics Committee of the Faculty of Medicine at our University and conducted according to the World Medical Association Declaration of Helsinki. Patients were excluded if they have other systemic autoimmune diseases, diabetes, hypertension, and a history of CVD. All clinical assessments were completed at one baseline study visit. SLE disease activity was assessed by Systemic Lupus Erythematosus Disease Activity Index-2000 (SLEDAI-2 K).^17^ Active disease was defined as a SLEDAI-2 K score ≥6 and Inactive disease SLEDAI-2 K score < 6. Body mass index (BMI) (kg/m2) and waist-to-hip ratio (WHR) were calculated. Lipid profiles and other laboratory investigations were done.

### Detection of Breg cells frequencies

**Figure 1.**
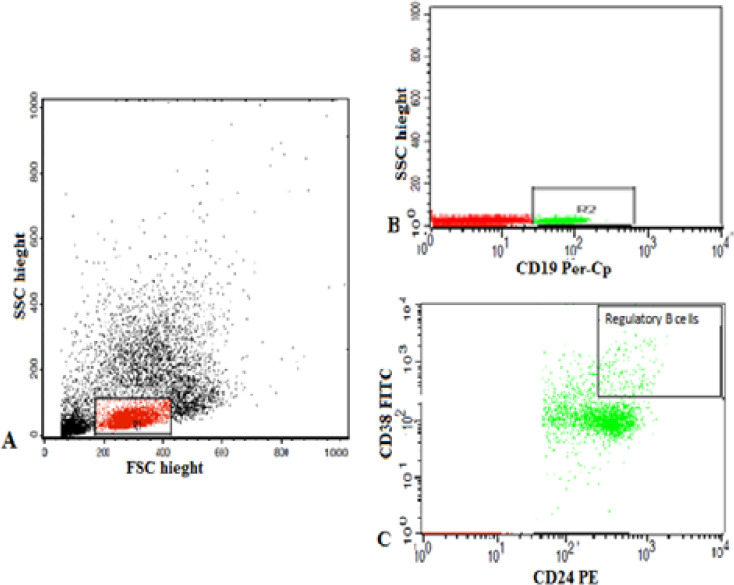
Flow cytometric detection of regulatory B cells **(a)** forward and side scatter plot was used to define the lymphocytes population (R1). **(b)** CD19+ cells (R2) were assessed within the lymphocyte population. **(c)** The expression of CD24 and CD38 was assessed in CD19+ cells to define CD19^+^CD24^high^CD38^high^ cells.

Evaluation of Breg cells frequencies by flow cytometry, circulating Breg cells were detected using FITC-conjugated-CD38, PE-conjugated-CD24 (BD Bioscience, USA), and PerCP-conjugated CD19 (BD Bioscience, USA).100 μl of blood sample was incubated with 10 μl of CD24, CD38, and CD19 for 20 minutes at 4 C in the dark. Following incubation, red blood cell lysis, washing, and analysis by FACS Calibur flow cytometry with Cell Quest software (Becton Dickinson Biosciences, USA) was done. An isotype-matched negative control was used for each sample. A Forward and side scatter histogram was used to define the lymphocytes. Then CD19+ B cells were gated. Then the expression of CD38 and CD24 on the CD19+B cells was revealed. Regulatory B cells were specified as CD19^+^CD24^high^CD38^high^ cells (**Figure 1**).

### Detection of subclinical atherosclerosis using cIMT measurement and CAC scoring: Assessment of the carotid intima-media thickness and plaque

All participants underwent carotid Doppler ultrasound examination using a Logic 3 ultrasound machine (GE medical systems, GE Healthcare, Milwaukee, WI, USA). The patient lay down in the supine or semi-supine position with the head slightly hyperextended and rotated 45° away from the side being examined. Higher-frequency linear transducer (≤7 MHz) was used to assess the intima-media thickness (IMT) and plaque morphology, as well as Doppler examination. The sonographer scanned the right and the left common carotid artery (CCA). The IMT of CCA was measured 1 cm distal to the carotid bifurcation in the posterior wall. The highest IMT among the measured segments studied on each side was registered. Multiple measurements of the IMT on both sides were taken and the mean value was recorded. We applied the European version where the IMT was considered “normal” when < 0.9 mm, “thickened” when the IMT was ≥ 0.9 mm, and when the thickness was > 1.3 mm it was a signal of atherosclerotic plaque.^18^

### Measurement of coronary artery calcification

All participants underwent coronary artery calcium scoring using a 64-slice CT scanner (General Electrics Light Speed VCT, Milwaukee, WI, USA). All scans were interpreted by the same cardiologist who was unaware of the patients’ clinical status at the time of interpretation. The coronary artery calcium scoring was calculated using the Agatston method.^19^ Semi-automated software was used to calculate the “Agatston score” after manual identification of calcified lesions in the coronary arteries. . In the absence of calcification, the score = zero. Minimal levels of identifiable calcification are scored (1–10), mild (11–100), moderate (101–400), and severe: >400.

### Statistical Analysis

Data was collected and analysed using SPSS (Statistical Package for the Social Science, version 20, IBM, and Armonk, New York). Continuous data were expressed in form of mean ± SD or median (range) while nominal data were expressed in form of frequency (percentage). A Student t-test was used to compare the mean of different two groups and an ANOVA test for more than two groups. Pearson correlation coefficient (r) was done to determine the correlation between quantitative variables indicated to the strength of correlation. Multivariate regression analysis was used to determine the independent risk factors. The receiver operating characteristic curve (ROC curve) was done to determine the diagnostic performance of Breg cells for prediction of SLE, active SLE, and atherosclerosis. The level of confidence was kept at 95%. P-value <0.05 was considered statistically significant.

## RESULTS

A total of 30 SLE patients 10 of them had APS and 23 healthy controls were analysed. 90% of patients were females. Demographics, clinical, and laboratory characteristics of SLE patients are presented in **Table 1**. Out of the studied patients, 23 (76.7%) patients had subclinical atherosclerosis and 18 (60%) patients had active disease. The right and left carotid intima-media thickness (cIMT) were higher in SLE patients than in the controls. Also, they had significantly higher mean cIMT than the controls (1.04± 0.25 vs. 0.91 ± 0.13 mm; P*=* 0.01). There was no statistical difference in mean cIMT between active and inactive SLE patients. **Table 2** describes that there was a statistically significant difference in B-lymphocytes percentages (%) between SLE patients with subclinical atherosclerosis and controls (P= 0.021). There was a highly statistically significant difference in Breg cells (%) between SLE patients with subclinical atherosclerosis and controls (P= 0.001). B-lymphocyte (%) was significantly higher in SLE with APS than in controls (P=0.001). Breg cells (%) were significantly lower in SLE patients with APS compared to controls (P=0.005). SLE patients with APS had significantly higher cIMT than controls. (P=0.050). **Table 3** demonstrated that there was no significant difference between B-lymphocytes (%) and Breg cells (%) of active SLE patients with and without subclinical atherosclerosis. **Table 4** showed that out of the studied patients, 9 (30%) patients had coronary artery calcification (CAC). It was noticed that patients with and without CAC had insignificant differences regarding Breg cells and mean cIMT. As detected in **Table 5**, there was no significant difference in B-lymphocytes (%) between SLE patients with CAC and increased cIMT (P= 0.833) or SLE patients with CAC and both increased cIMT and CAC. (P= 0.151). B-lymphocytes (%) were significantly higher in SLE patients with both increased cIMT and CAC compared to those with increased cIMT alone. (P= 0.030). No significant difference in Breg cell (%) between the three groups**.** SLE patients with APS had an insignificant negative correlation of Breg cells (%) with disease activity, cIMT, and CAC (*r* = −0.028, −0.233, −0.124) respectively. Correlations of Breg cells (%), Mean cIMT, and SLEDAI-2 K score with different parameters were reported in **Table 6**. Diagnostic accuracy of Breg cells in the prediction of SLE, active SLE, and atherosclerosis was presented in **Table 7**. In multivariate regression analysis for the prediction of disease activity It was noticed that the predictors were long disease duration (OR=3.43, 95% CI= 3.09-6.88; P= 0.03) and low Breg cells (OR=1.76, 95% CI= 1.21- 2.85; P= 0.01) (**Table 8**). In multivariate regression analysis for predicting thickened cIM, the predictor was coronary artery calcification (OR=1.78, 95% CI= 1.33-3.56; P= 0.04) (**Table 9**).

**Table 1.** Demographics, clinical, laboratory, and medications characteristics of SLE patients.

**Variables n%, mean ± SD or median (range)**	**n= 30**
**Age (years)**	32.60 ± 8.34
**Gender**	
**Male**	3 (10%)
**Female**	27 (90%)
**Age of onset (years)**	28.27 ± 7.60
**Duration (years)**	5.0 (1.0-14.0)
**Body mass index (kg/m^2^)**	26.87 ± 7.029
**Arthritis**	21 (70%)
**Mucocutaneous**	24 (80%)
**manifestations**	
**Renal manifestations**	19 (63.3)
**Cardiopulmonary**	7 (23.3%)
**manifestations**	
**SLEDAI-2 K score**	6.0 (0.0-22.0)
**mean cIMT**	1.04 ± 0.25
**Haemoglobin (g %)**	11.54 ± 1.45
**Leucocytes (x 106/ml)**	5.32 ± 1.75
**Platelets (x 10^6^/ml )**	232.83 ± 75.83
**Urinary protein (mg/24h)**	1331.07 ± 345.99
**Antiphospholipid**	10 (33.3)
**antibodies**	
**DMARDs**	30 (100%)
**Steroids**	18 (60%)
**Anticoagulant**	9 (30%)
**Antiaggregant**	10 (33.3)

SLEDAI-2 K: Systemic Lupus Erythematosus Disease Activity Index-2000; cIMT: carotid intima-media thickness; DMARDs: Disease-modifying antirheumatic drugs; SD: standard deviation; n: number.

**Table 2.** B-lymphocyte and Breg cells (%) in SLE patients with subclinical atherosclerosis, SLE patients with APS and controls.

	**SLE with atherosclerosis (n= 23)**	**Controls (n= 23)**	**P-value**	**SLE with APS (n= 10)**	**Controls (n= 23)**	**P-value**
**B-lymphocytes (%) mean (SD)**	17.91 ± 5.60	13.75 ± 2.42	0.021	19.51 ± 6.08	13.49 ± 3.08	0.001
**Breg cells (%) mean (SD)**	2.36 ± 0.70	3.52 ± 1.60	0.001	2.23 ± 0.68	3.66 ± 1.41	0.005

SLE: Systemic lupus erythematosus; Breg cells: Regulatory B cells; SD: standard deviation; APS: antiphospholipid syndrome; n: number.

**Table 3.** B-lymphocytes and Breg cells (%) in active SLE patients with and without subclinical atherosclerosis.

	**Active SLE with atherosclerosis (n= 11)**	**Active SLE without atherosclerosis (n= 7)**	**P-value**
**B-lymphocytes (%) mean (SD)**	18.16 ± 5.56	14.86 ± 3.40	0.180
**Breg cells (%) mean (SD)**	2.22 ± 0.59	2.43 ± 0.66	0.482

SLE: Systemic lupus erythematosus; Breg cells: Regulatory B cells; SD: standard deviation; n: number.

**Table 4.** Breg cells (%) and mean cIMT based on CAC.

	**SLE With CAC (n= 9)**	**SLE Without CAC (n= 21)**	**P-value**
**Breg cells (%) mean (SD)**	2.63 ± 0.79	2.26 ± 0.64	0.190
**Mean cIMT (mm) mean (SD)**	1.03 ± 0.32	1.05 ± 0.24	0.885

SLE: Systemic lupus erythematosus; Breg cells: Regulatory B cells; cIMT: carotid intima-media thickness; CAC: coronary artery calcification; SD: standard deviation; n: number.

**Table 5.** B-lymphocytes, Breg cells (%) based on CAC and increased cIMT.

	**CAC (n= 4)**	**Increased cIMT (n= 14)**	**Both (n= 5)**	**P-value^1^**	**P-value^2^**	**P-value^3^**
**B-lymphocytes (%) mean (SD)**	15.78 ± 4.49	16.29 ± 4.11	22.44 ± 7.15	0.833	0.151	0.030
**Breg cells (%) mean (SD)**	2.48 ± 0.73	2.22 ± 0.60	2.75 ± 0.89	0.487	0.639	0.157

Breg cells: Regulatory B cells; cIMT: carotid intima-media thickness; CAC: coronary artery calcification; SD: standard deviation; n: number.

P-value^1^: Comparison of CAC and increased cIMT;

P-value^2^: Comparison of CAC and with both increased cIMT and CAC:

P-value^3^: Comparison of cIMT and both increased cIMT and CAC.

**Table 6.** Correlation of Breg cells (%), mean cIMT and SLEDAI-2 K score with different parameters in SLE patients.

	Breg (%)		Mean cIMT		SLEDAI-2 K score	
	r-value	P-value	r-value	p-value	r-value	P-value
Age (years)	**-0.065**	**0.732**	**0.276**	**0.140**	**-0.001**	**0.994**
BMI	**-0.211**	**0.262**	**0.004**	**0.983**	**0.138**	**0.467**
WHR	**-0.290**	**0.120**	**0.080**	**0.673**	**0.215**	**0.253**
B-lymphocytes (%)	**-0.197**	**0.296**	**0.187**	**0.323**	**0.131**	**0.455**
Duration of disease (years)	**0.154**	**0.418**	**-0.026**	**0.893**	**0.118**	**0.536**
Age of onset (years)	**-0.111**	**0.560**	**0.303**	**0.104**	**-0.089**	**0.639**
Haemoglobin (g %)	**-0.152**	**0.423**	**0.142**	**0.453**	**-0.357**	**0.05**
Leucocytes (x 10^6^/ml )	**0.067**	**0.726**	**0.017**	**0.930**	**0.149**	**0.432**
Platelets (x 10^6^/ml )	**-0.055**	**0.772**	**-0.139**	**0.465**	**-0.026**	**0.891**
Triglyceride	**-0.190**	**0.314**	**0.035**	**0.810**	**0.178**	**0.346**
Cholesterol	**-0.089**	**0.621**	**0.038**	**0.756**	**0.146**	**0.442**
HDL	**0.133**	**0.492**	**-0.082**	**0.647**	**-0.003**	**0.988**
LDL	**-0.129**	**0.506**	**0.065**	**0.672**	**-0.062**	**0.743**
ESR	**0.155**	**0.412**	**0.185**	**0.327**	**0.069**	**0.718**
SLEDAI-2 K	**-0.006**	**0.965**	**0.143**	**0.467**	**-**	**-**
Mean cIMT	**-0.028**	**0.905**	**-**	**-**	**0.143**	**0.467**

Breg cells: Regulatory B cells; cIMT: carotid intima-media thickness; SLEDAI-2 K: Systemic Lupus Erythematosus Disease Activity Index-2000; BMI: Body mass index; WHR: waist-to-hip ratio; HDL: High- density lipoprotein; LDL: low-density lipoprotein; ESR: Erythrocyte sedimentation rate.

**Table 7.** Diagnostic accuracy of Breg cells in the prediction of SLE, active SLE, and atherosclerosis.

**Indices**	**SLE**	**Active SLE**	**Atherosclerosis**
**Sensitivity**	87%	94%	21%
**Specificity**	70%	33.3%	63%
**Positive predictive value**	79%	68%	50%
**Negative predictive value**	80%	80%	32%
**Accuracy**	79.25	66.67	36.67
**Area under the curve**	0.81	0.54	0.51
**Cut off point**	< 3.01	< 2.9	< 1.73
**P- value**	0.01	0.03	0.67

SLE: Systemic lupus erythematosus; Breg cells: Regulatory B cells.

**Table 8.** Multivariate regression analysis for the prediction of disease activity.

**Variables**	**Odds ratio**	**95% confidence interval**	**P- value**
**Age**	0.45	0.11- 1.99	0.09
**Sex**	0.98	0.33- 3.87	0.11
**Age of onset**	1.45	1.1- 3.11	0.08
**Duration**	3.43	3.09- 6.88	0.03
**Coronary calcification**	1.09	0.89- 1.65	0.34
**Breg cell**	1.76	1.21- 2.85	0.01

Breg cells: Regulatory B cells.

**Table 9.** Multivariate regression analysis for the prediction of thickened cIMT.

**Variables**	**Odd’s ratio**	**95% confidence interval**	**P- value**
**Age**	0.34	1.09- 4.57	0.93
**Sex**	1.91	0.87- 1.07	0.19
**Age of onset**	0.30	0.43- 2.11	0.88
**Duration**	0.98	0.09- 1.08	0.73
**Coronary calcification**	1.78	1.33- 3.56	0.04
**Breg cell**	1.09	1.01- 1.89	0.41

Breg cells: Regulatory B cells; cIMT: carotid intima-media thickness.

## DISCUSSION

Increased atherosclerosis in SLE patients is assumed to be an outcome of a mixture of traditional risk factors, inflammation, and immune dysregulation.^20^ Subclinical atherosclerosis is an early predictor of atherosclerotic burden, and learning of it may prevent the progression of CVD.^21^ Within the current study, we observed a high frequency of subclinical atherosclerosis in SLE patients (76.7%), and 30% of patients had coronary artery calcification (CAC). These results were almost like previous studies where CAC in young, asymptomatic patients with SLE had signs of coronary atherosclerosis in 28-30%.^22,23^ Carotid plaques in SLE are higher 2.4 times than in the general population with 5.6 times more in patients <40 years.^5^ Increase the risk for acute myocardial infarction presents in young SLE females.^24^ CAC appears to be more in younger SLE patients than controls.^25^ Considering the age-related risk, a high prevalence of atherosclerosis in middle-aged SLE patients existed. ^26^ Compatible with mentioned above results, our atherosclerotic patients had young age. In our study, patients had significantly higher mean cIMT as compared to the controls, also patients with and without CAC had insignificant differences regarding Breg cells and mean cIMT. There was a significant increase of cIMT in SLE patients than in the controls in the study of Tyrrell et al.^27^ Elevation of cIMT was found in 28% of patients with SLE.^28^ Divided from our work, Gallelli et al. in their study didn’t have significant differences between the cIMT and controls.^29^ We observed no statistical difference in mean cIMT between active and inactive SLE patients that believe the study of Sazliyana et al.,^30^ while other studies had a significant correlation between cIMT and disease activity.^31,32^ The contribution of B cell activation in the pathogenesis of lupus and atherosclerosis referred to the importance of B cell-mediated effects on lupus-related atherosclerosis.^33^ B cells seem to exacerbate atherosclerosis by utilizing antibodies against oxLDL epitopes and also the promotion of an atherogenic cytokine profile.^34^ SLE B cell dysregulation can result in T cell activation, dendritic cell recruitment, induction of Th1 and Th17 cells, and inhibition of Treg.^35^ Breg in atherosclerosis has a tendency of atheroprotective effects^15^ by their production of IL-10, Breg may have anti-atherogenic properties and reduce autoimmunity.^36^ Moreover, the adoptive transfer of a Breg subset from Apoe-/- mice, leads to a decrease in atherosclerosis development.^15^ Several studies demonstrated that SLE patients were characterized by a numerical and functional deficiency in circulating Bregs, the defect was related to the failure of immature CD19^+^CD24^high^CD38^high^ B cells to differentiate into Bregs.^33,37^ Others showed that the proportion of CD19^+^CD24^high^CD38^high^ Breg cells was expanded in SLE, compared with healthy controls.^14,38^ We aimed within the current study to analyse this hypothesis in our patients and we found the mean of B-lymphocytes was higher while the mean of Breg cells was less than controls in SLE patients with subclinical atherosclerosis. We also observed that the mean of B-lymphocytes was higher in active SLE patients with subclinical atherosclerosis than in active SLE patients without atherosclerosis and there was no significant difference between Breg cells in both groups. Additionally, B -lymphocytes were significantly higher in SLE patients with both CAC and increased cIMT compared to those with increased cIMT alone with no significant difference in Breg cell percentage between the three groups. This might explain the importance of B cells within the pathogenesis of atherosclerosis in SLE and therefore the probability of increased risk of cardiovascular complications. Liu et al. concluded in their study, that CD19^+^CD24^high^CD38^high^ Breg is numerically and functionally deficient in coronary artery disease patients.^37^ Our detailed analysis revealed the presence of an insignificant negative correlation between Breg cells with age, age of onset, lipid profile, disease activity, and mean cIMT. This agrees with the previously mentioned that the immune dysregulation of lupus is important in plaque progression and vascular complications.^36^ In SLE, disease duration is related to increased CVD burden. Moreover, long duration has been related to CAC and therefore the formation of atherosclerotic plaques.^5^ However, this differed from our results that exposed there was a negative correlation between cIMT and disease duration, which can give attention to the occurrence of subclinical atherosclerosis in the earlier stage of SLE. The cIMT within the SLE group had an insignificant positive correlation with age, age of onset, lipid profile, and disease activity. Many studies discussed the relation between atherosclerotic plaque and disease activity, a study found that active disease was predictive of carotid plaque and thickened mean IMT,^28^ and Manzi et al. mentioned that the SLE activity was inversely associated with the presence of atherosclerotic plaque.^39^ Another study didn’t show a correlation between cIMT and disease activity.^40^ In SLE, dyslipidaemia is characterised by increased LDL and triglycerides and decreased HDL.^39^ SLE offers an appropriate situation for LDL oxidation and therefore the formation of immunogenic oxLDL.^34^ HDL is considered atheroprotective, but in chronic inflammation like SLE, HDL can lose its anti-inflammatory effects and turn into pro-inflammatory (piHD).^41^ As a result, it fails to efficiently participate in cholesterol efflux and allows for the oxidation of LDL. The piHDL form is related to the progression of carotid plaques and IMT in SLE patients.^41^ Ronda et al. said that the antiatherogenic property of HDL is impaired in SLE.^42^ Increase the development of CVD by up to 17-time in SLE patients having piHDL denoting that HDL is a risk factor of atherosclerosis in SLE .^43^ A study found that thirty-five percent of adult SLE patients have abnormal lipoprotein profiles and increases to 60% during the next 3-year.^44^ Breg cells had 70% sensitivity and 87% specificity for diagnosing SLE (P=0.01) and 94% sensitivity and 33.3% specificity for the prediction of active SLE (P=0.03) Therefore, Breg cells are considered to be a good predictor of disease activity and diagnosis of SLE. In the multivariate regression analysis, long disease duration had 3.43 times and low Breg cells had 1.76 times as a predictor of disease activity. While coronary calcification had 1.78 times as a predictor of thickened cIMT.

In a high percentage of APS patients, silent myocardial ischemia, pulmonary pressure elevation, and coronary atherosclerosis are found and associated with APA.^45^ Antiphospholipid antibodies may contribute to the event of atherosclerosis in SLE. ^46^ During young adulthood, APA positivity may be a risk factor for subsequent sub-clinical atherosclerosis and might play a task in the pathogenesis of atherosclerosis.^47^ APA contributes to thrombosis formation by activating different cell types as endothelial cells, monocytes, platelets, neutrophils, and fibroblasts.^48^ We found that Breg cells (%) were significantly lower in SLE patients with APS compared to healthy controls. Breg cells (%) in SLE patients with APS had an insignificant negative correlation with disease activity, cIMT, and CAC. These data may denote the atheroprotective role of the Breg cells in APS patients. Within the current study, 30% of SLE patients with secondary APS had CAC. Also, SLE patients with secondary APS had significantly higher cIMT than healthy controls this can be accepted as true with the study of Di Minno et al.^49^ From the foregoing, CV risk factors, subclinical atherosclerosis, effects of SLE disease, APA, and Breg cells all at once may exert an influence on the outcome of SLE. We’ve got limitations because it was a cross-sectional study with a small number of patients. We recommend carrying out more longitudinal research with a larger sample size and a study of other subsets of Breg cells. This text provides insight into the important role of Breg cells in SLE patients with subclinical atherosclerosis and secondary APS. Hopefully, in the future, Breg cells will take an opportunity as a treatment for both SLE and atherosclerosis.

## CONCLUSION

SLE patients had a high frequency of subclinical atherosclerosis, those and patients with secondary APS had a high risk of plaque formation. We found a contribution of Breg cells in patients of SLE with subclinical atherosclerosis. Breg cells (%) were numerically decreased in SLE patients with subclinical atherosclerosis and with secondary APS. Breg cells are considered to be a good predictor of diagnosis of SLE.
